# Behavioral Reversion and Dark–Light Choice Behavior in Workers of the Red Wood Ant *Formica polyctena*

**DOI:** 10.1007/s10905-015-9496-2

**Published:** 2015-04-02

**Authors:** Beata Symonowicz, Maria Kieruzel, Anna Szczuka, Julita Korczyńska, Andrzej Wnuk, Paweł Jarosław Mazurkiewicz, Michał Chiliński, Ewa Joanna Godzińska

**Affiliations:** 1 Department of Neurophysiology Laboratory of Ethology, Nencki Institute of Experimental Biology, Pasteur St 3, 02-093 Warsaw, Poland; 2 College of Inter-Faculty Individual Studies in Mathematics and Natural Sciences, University of Warsaw, Żwirki i Wigury St. 93, 02-089 Warsaw, Poland

**Keywords:** Behavioral reversion, nurse, forager, reverted nurse, dark–light choice, *Formica polyctena*

## Abstract

Social insect workers usually start adult life from intranidal tasks and then switch to extranidal activities, but this process may be reversed: foragers may switch again to intranidal brood care. The transition forager – reverted nurse is known as the behavioral reversion. Ant foragers are known to avoid illuminated zones less strongly than intranidal workers, but illumination responses of reverted nurses were so far never investigated. We compared dark–light choice behavior of three classes of workers of the red wood ant *Formica polyctena*: nurses, foragers and reverted nurses. Sets of ten ants belonging to the same class were tested in “double nests” made of two interconnected test tubes, one kept in darkness and the other exposed to light. The number of ants present in the illuminated zone of each nest (n_i_) was recorded on 10 sample points at 30 min intervals. The values of n_i_ were lower in nurses than in foragers and reverted nurses and decreased as a function of time in all three groups. Nurses differed from foragers with respect to the dynamics of dark–light choice behavior, but reverted nurses did not differ in that respect either from nurses, or from foragers. Reverted nurses and foragers did not differ significantly from each other with respect to the overall level of avoidance of illuminated zone, nor with respect to the dynamics of dark–light choice behavior. This implies that behavioral reversion is not accompanied by the return of illumination responses of workers of *F. polyctena* to the state characteristic for nurses.

## Introduction

Social insect workers as a rule start their adult life from intranidal tasks and then switch to extranidal activities (Otto 1958; Dobrzańska 1959; Lenoir 1979a, 1987; Hölldobler and Wilson 1990, 2009; Fénéron et al. 1996). Foragers may, however, switch again to intranidal brood care and become the so called reverted nurses. This phenomenon is known as the behavioral reversion and may be induced by specific modifications of the social context, in particular by the exposure of foragers to brood in absence of younger workers acting as nurses (Ehrhardt 1931; Dobrzańska 1959; Lenoir 1979a, 1987; Robinson et al. 1992; Page and Amdam 2007; Amdam 2011; Herb et al. 2012; Kuszewska and Woyciechowski 2013).

Behavioral reversion was investigated in the most detailed way in the honeybee (*Apis mellifera* L). Extensive research devoted to that phenomenon revealed that honeybee behavioral reversion is not limited to the induction of brood care behavior, but is also accompanied by other modifications of worker behavior (Bloch and Robinson 2001; Behrends et al. 2007; Baker et al. 2012) and by the reversal of numerous other profound phenotype modifications accompanying worker transition from intranidal to extranidal tasks. In particular, it is accompanied by the regeneration of the hypopharyngeal and wax glands, the increase in the diameter of the ovarioles, and the reversal of modifications of hemolymph titres of juvenile hormone and vitellogenin, of age-related decline in immunity (immunosenescence), of changes of biogenic amine levels in specific brain structures, and of some (but not all) modifications of the whole-body protein profile, gene expression, and gene methylation levels (Rösch 1930; Free 1965; Robinson et al. 1992, Amdam and Page 2005; Amdam et al. 2005; Wolschin and Amdam 2007; Münch et al. 2008; Amdam 2011; Herb et al. 2012; Kuszewska and Woyciechowski 2013; Margotta et al. 2013).

Behavioral reversion was also investigated in various species of ants (Ehrhardt 1931; Weir 1958; Dobrzańska 1959; Lenoir 1979a, b; Sorensen et al. 1984; McDonald and Topoff 1985; Wnuk et al. 2011; Korczyńska et al. 2014). Phenotype modifications accompanying ant behavioral reversion are, however, much less known than those documented in the honeybee. Several authors also pointed out that honeybee and ant behavioral reversion may represent fundamentally different phenomena (Lenoir 1979a; Sorensen et al. 1984). In particular, whereas in the honeybee phenotype modifications accompanying behavioral reversion develop in a relatively slow and gradual way (Rösch 1930; Robinson et al. 1992; Huang and Robinson 1996), in ants induction of brood care may occur very rapidly, within 24 h, which argues against the exocrine control of that process (Lenoir 1979a; Sorensen et al. 1984; McDonald and Topoff 1985; Wnuk et al. 2011). Moreover, in the case of ants the question of the expression of brood care behavior is more complex than in the case of the honeybees and requires still much clarification. In many ant species foragers, and not nurses, are more attracted to brood found outside of the nest and show higher readiness to retrieve it to the nest (Weir 1958; Lenoir 1977, 1981). Older ant workers and/or foragers may also engage in intranidal brood care, sometimes even during their whole life (Lenoir 1979a, 1987; Calabi et al. 1983; Déjean and Lachaud 1991; Retana and Cerdá 1991; Godzińska et al. 1999, 2001; Seid and Traniello 2006; Muscedere et al. 2009), and the same individuals may engage in both nursing and foraging behavior (Sorensen et al. 1984; Seid and Traniello 2006; Muscedere et al. 2009). These findings suggest that the notion of the transition nurse – forager should rather be replaced in ants by the notion of age-related expansion of behavioral repertoire (Seid et al. 2005; Seid and Traniello 2006; Muscedere et al. 2009, 2013). In light of the differences between honeybee and ant behavioral reversion it seems highly probable that further research on ant behavioral reversion may discover yet other phenomena unknown and/or absent in the honeybee.

Together with earlier studies of our team (Wnuk et al. 2011; Korczyńska et al. 2014), our present study belongs to a series of experiments carried out to shed more light on behavioral, neurochemical and anatomical correlates of ant behavioral reversion. In particular, we were interested if ant behavioral reversion consists solely of the induction of intranidal brood care behavior, or involves also other modifications of worker behavior. We decided to study the impact of behavioral reversion on dark–light choice behavior of ant workers, as important modifications of that behavior taking place in the course of worker ontogeny were already documented by several studies (Rosengren 1971, 1977; Wehner et al. 1972; Rosengren and Sundström 1987). In particular, extranidal workers were reported to avoid illuminated zones less strongly than intranidal ones (*Formica rufa* L.; Rosengren 1971), and to approach white silhouettes rather than dark ones (*Cataglyphis bicolor* Fabricius; Wehner et al. 1972). Task-related differences in dark–light choice behavior of workers of *C. bicolor* were also found to be largely reversible under the influence of worker exposure to specific illumination conditions (Wehner et al. 1972). However, dark–light choice behavior of the reverted ant nurses was so far never investigated.

In the present study we compared dark–light choice behavior of nurses, foragers and reverted nurses of the red wood ant (*Formica polyctena* Först.). We choose that species, as phenotype modifications accompanying behavioral reversion were already investigated in *F. polyctena* (Wnuk et al. 2011), and as task- and age-related modifications of dark–light choice behavior were already reported in *F. polyctena* and a closely akin species *F. rufa* (Rosengren 1971, 1977; Rosengren and Sundström 1987). On the basis of these literature data, we expected that foragers of *F. polyctena* will avoid light less strongly than nurses. However, we focused our attention above all on the impact of both past and present behavioral specialization on dark–light choice behavior of the reverted nurses, and on the question of reversibility of developmental modifications of that behavior.

## Methods

### Subjects

Workers used in the experiment were collected from a colony of *Formica polyctena* located in the mixed pine forest close to the village Wólka Radzymińska near Warsaw in Central Poland (52°25’57” N, 21°5’8” E). Nurses and foragers were collected separately on 15th April 2008 and 28th May 2008, respectively. In spring queens, nurses and brood of *F. polyctena* can be easily collected from the top parts of their mounds. A large colony fragment consisting of about 10–12 thousands of workers including nurses, abundant brood (eggs and larvae) and numerous (about 730) dealated queens was collected from the top of the nest mound together with the nest material. Foragers (about 7000) were collected from the trails at a distance of 3–4 m from the mound. The collected ants were transferred to laboratory and housed in artificial nests.

The ants collected from the mound were housed in a large nest composed of three large (38 cm × 30 cm × 15 cm) rectangular open Perspex containers connected by means of narrow tubes (Nest A). The walls of the nest were coated with Fluon® (PTFE), a substance providing silky smooth surface and, hence, commonly used in myrmecological research to prevent the ants from escaping from artificial nests. The floor of the nest was covered by a thin layer of fine sand. Two out of three interconnected boxes contained artificial brood chambers made of numerous large glass test tubes filled partly with water trapped in by means of a cotton plug to provide a humidity gradient allowing the ants to choose the preferred humidity conditions. The tubes were covered from above by a sheet of aluminum foil to assure darkness. The third box served as a foraging area. The ants had ad libitum access to water (provided in small Petri dishes filled with moist cotton) and to carbohydrate and protein food (honey mixed with crushed apple and with sand added to make the mixture less sticky and pieces of house crickets killed by freezing and allowed to thaw at room temperature).

Foragers taken from the trails were housed in a similar nest (Nest B) made of two smaller (30 × 20 × 15 cm) interconnected containers. One of these boxes contained test tubes serving as artificial nest chambers, the other served as the foraging area. A week after their collection in the field foragers were divided into two groups, each counting approximately 3500 individuals. One of these groups was transferred to yet another nest (Nest C) in order to be subjected to the induction of behavioral reversion. The Nest C was composed of a single box (30 cm × 20 cm × 15 cm) containing several artificial nest chambers covered with aluminum foil to assure darkness. The ants were fed in the same way and kept in the same conditions as the ants from the nests A and B. The second group of foragers continued to be housed in the Nest B. All the nests were kept at a stable ambient temperature (24 °C) and relative humidity of the air (48–58 %) and exposed to a natural rhythm of daylight and darkness supplemented by artificial white light illumination delivered at 12:12 LD.

### Behavioral Reversion

The induction of behavioral reversion was carried out on 15–23 June 2008. On each day a relatively large amount (about 100 ml) of worker pupae taken from the nest A was gently placed on the floor of the nest C. After about 24 h, when all the pupae were already transported by the ants to test tubes acting as artificial nest chambers, the tubes were taken out and their contents (pupae and ant workers) were gently transferred to a cylindrical glass container (10 cm high, inner diameter 22.5 cm) with the walls covered with Fluon®. The workers which moved away from the brood pile were put again in the nest C. All newly eclosed callows were also removed to avoid possible future difficulties in distinguishing them from the reverted nurses. The pupae and the workers engaged in brood care (usually about 400 individuals) were then placed in a new nest, also made of a single box (30 cm × 20 cm × 15 cm). This procedure was repeated on four successive days (16–19 June 2008). As a consequence, we created a series of nests (D1-D4), each of them containing the reverted nurses that switched to brood care on a particular day. Each nest contained 6 test tubes equipped with water reservoirs and covered by aluminum foil. Drinking water and food was provided in a similar way as in the case of the remaining nests. The state of the nests was monitored daily. We removed newly eclosed callows and, if necessary, added new worker pupae taken from the nest A to maintain an approximately constant volume of brood. The reverted nurses were tested after 7 days from the moment of the creation of the nest (D1-D4) to which they were transferred after having been found to engage in brood care in the nest C.

### Selection of Workers for Behavioral Tests

We tested dark–light choice behavior of three groups of ants: nurses, foragers and reverted nurses. Nurses and reverted nurses were taken, respectively, from among workers present in the brood chambers of the nest A housing the main colony fragment collected from the mound, and the nests from the series D1-D4 housing groups of reverted nurses created a week earlier. In each case the tubes acting as brood chambers were taken out of the nest and their contents were gently transferred to a cylindrical container (10 cm high, inner diameter 23 cm) with the walls covered with Fluon®. The ants that walked away from the brood pile were removed. The remaining ants were left to settle during 30 min. After that time only the ants that settled on the brood pile in direct contact with the pupae were selected for the dark–light choice tests.

Foragers were selected from among workers present in the foraging area of the nest B. They were captured by means of a forceps and transferred directly to a cylindrical container similar to that used in the process of the selection of both types of nurses. They were then left to settle during 30 min and then started to be transferred to the nests used in the dark–light choice tests.

### Dark–Light Choice Test

**Fig. 1:**
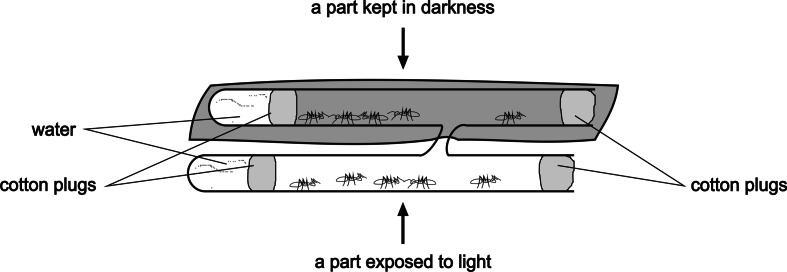
The double nest used in the tests investigating dark–light choice behavior of workers of the red wood ant *Formica polyctena*. A set of 10 workers was tested simultaneously. At the start of the test an equal number of workers (*n* = 5) was put in each arm of the nest and then the number of workers present in the illuminated half of the nest was recorded at 30 min intervals

Dark–light choice behavior of workers of *F. polyctena* was tested by means of a bioassay proposed by Korczyńska and Godzińska (1995, 2000). That bioassay involved the use of the so called double nests (Fig. 1) composed of two large glass test tubes (22 cm long, inner diameter 1.7 cm) connected by a narrow passage (4.5 cm long, inner diameter 0.5 cm). Each tube contained a water reservoir (2 cm long) kept in by a humid cotton plug (1.5 cm long). One half of the nest was kept in darkness (covered by several layers of dark cloth) and the other half was exposed to daylight supplemented with artificial white light provided by electric bulbs and the lamp “Fotovita FV-10” (manufacturer: ULTRA-VIOL sp. j.). The illumination level measured close to the upper surface of the double nests was set at 1000 ± 100 lx. The double nests used to test the ants from various groups were spatially interspersed to reduce as far as possible the impact of these small differences in illumination level on ant behavior. During the whole experiment ambient temperature ranged from 24 to 25 °C . The temperature measured in the illuminated zones of the double nests was only very slightly higher (about 0.05 °C) than the temperature measured in their dark parts.

At the start of the test a group of 10 workers belonging to the same class (nurses, foragers or reverted nurses) was placed in the double nest (five workers in each arm) and then the number of workers present in the illuminated half of the nest was counted at 30 min intervals. Altogether, 10 sample points were taken for each nest. The first measurement was carried at 10.30 a.m., about 30 min after the moment when all the workers tested on this particular day were already placed in the double nests. All manipulations necessary to prepare the experimental situation were carried out in parallel by several persons to minimize the differences between the time spent by the ants in the double nests prior to the initial measurement. Ants from various experimental groups were placed in the double nests in parallel.

The tests were carried out on four successive days (23–26 June 2008). On each day we tested an equal number of the double nests per group (9 nests per group on the first day, 7 nests per group on the second day, and 12 nests per group on each of the last 2 days of the experiment). Finally we analyzed the data obtained for 40 sets of 10 ants for each experimental group. Only the tests during which no worker died were taken into account in the analysis of the data. Worker mortality proved to be very low: only 3 foragers and 1 nurse were found dead after the end of the test.

### Quantification and Analysis of Ant Behavior

The main dependent variable analyzed in this study (n_i_) was the number of ants present in the illuminated zone of each nest on a particular sample point (1–10). The dependence of that variable on ant group (3 groups: nurses, reverted nurses and foragers) and on time from the start of the test (10 successive sample points) was analyzed by means of Repeated Measures ANOVA with group as a between-subject variable, time as a within-subject variable and the interaction between these two variables. The overall analysis of the data obtained for all three groups was followed by pairwise comparisons (nurses vs foragers, nurses vs reverted nurses and reverted nurses vs foragers) also carried out by means of Repeated Measures ANOVA with probabilities adjusted by means of Bonferroni correction. All these analyses were carried out by means of the software SPSS.

## Results

**Fig. 2:**
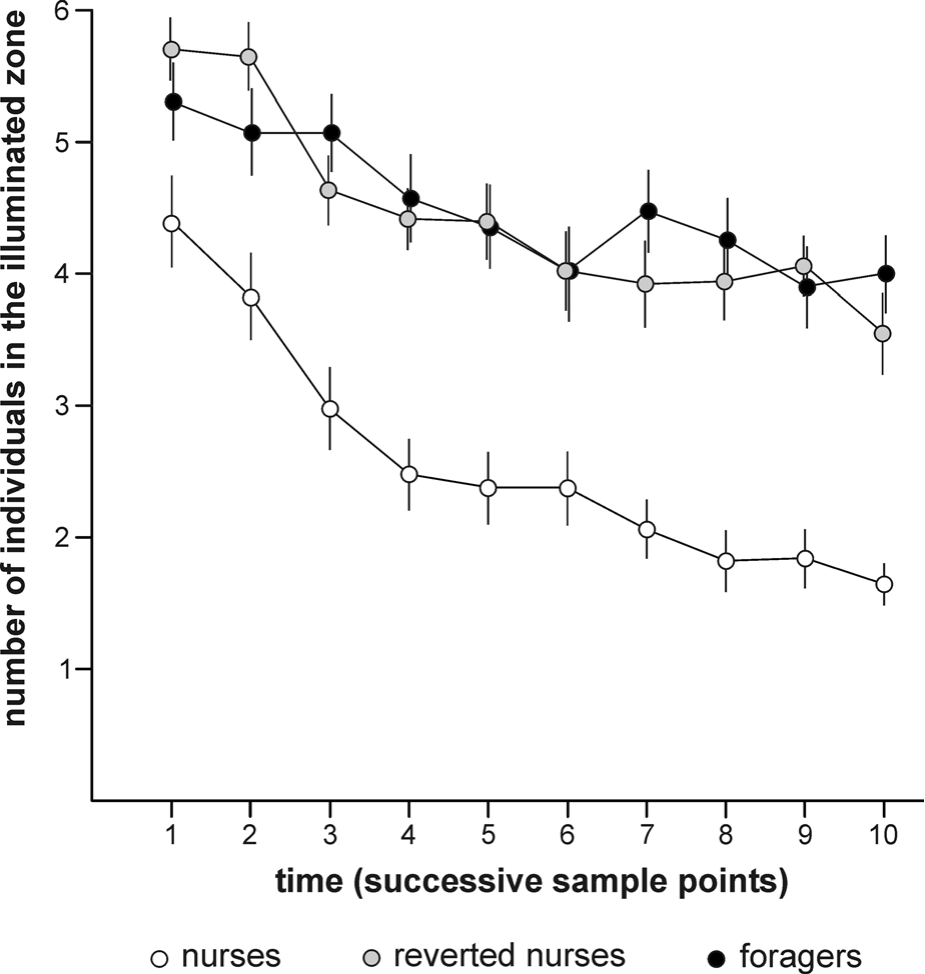
Values (mean ± SE) of the variable n_i_ (number of individuals present in the illuminated zone of the dark–light choice nest) obtained for three groups of workers of the red wood ant *Formica polyctena* (nurses, reverted nurses and foragers) on 10 successive sample points separated by 30 min intervals. The first sample point took place after 30 min from the start of the test session. Each experimental group consisted of 40 sets of 10 workers tested together in the same dark–light choice nest

The behavior of workers of *F. polyctena* tested in dark–light choice nests depended both on worker group (nurses, reverted nurses and foragers) and on time from the start of the test (ten sample points separated by 30 min intervals). The values of the variable n_i_ denoting the number of ants staying in the illuminated zone of the dark–light choice nest were significantly higher in the case of nurses than in the case of both classes of ants that had already passed the transition from intranidal to extranidal tasks, and decreased as a function of time in all three groups (Fig. 2). The overall Repeated Measures ANOVA applied to compare the values of n_i_ obtained for all three ant groups revealed a highly significant effect of the between-subject effect „group” (*F*_*2,117*_ = 27,297, *P <* 0.00001), a highly significant effect of the within-subject effect „time” (*F*_*9,1053*_ = 31.303, *P* < 0.00001), and a significant interaction „group” x „time” (*F*_*18,1053*_ = 1.721, *P* = 0.031). This last result implies that the dynamics of dark–light choice analyzed as a function of time from the start of the test differed between the compared groups.

Subsequent pairwise comparisons (nurses vs foragers, nurses vs reverted nurses and reverted nurses *vs* foragers) also carried out by means of Repeated Mesures ANOVA yielded more precise information about the differences between the tested ant groups.

The comparison nurses vs foragers discovered highly significant effects of „group” (*F*_*1,78*_ = 37.75, *P* < 0.00001) and „time” (*F*_*9,702*_ = 21.383, *P* < 0.00001) and a significant interaction „group” x „time” (*F*_*9,702*_ = 2.974, *P =* 0.006). Such a constellation of significant effects implies that nurses and foragers differed not only with respect to the overall level of avoidance of illuminated zones, but also with respect to the dynamics of their dark–light choice behavior (changes of n_i_ as a function of time from the start of the test). Nurses were withdrawing from illuminated zones more rapidly than foragers.

The comparison of nurses with reverted nurses discovered highly significant effects of „group” (*F*_*1,78*_ = 44.527, *P* < 0.00001) and „time” (*F*_*9,702*_ = 30.944, *P* < 0.00001), but interaction „group” x „time” was not significant (*F*_*9,702*_ = 0.822, *P =* 1.000). This implies that although the overall level of avoidance of illuminated zones differed between nurses and reverted nurses, the dynamics of dark–light choice behavior did not differ significantly between these two ant groups.

Lastly, the comparison of reverted nurses with foragers discovered no significant effect of „group” (*F*_*1,78*_ = 0.061, *P* = 1.000), highly significant effect of „time” (*F*_*9,702*_ = 13.6604, *P* < 0.00001), and no significant interaction „group” x „time” (*F*_*9,702*_ = 1.392, *P =* 0.564). This implies that neither the overall level of avoidance of illuminated zones, nor the dynamics of dark–light choice differed between reverted nurses and foragers.

The results of all ANOVA analyses taken together demonstrated that nurses avoided illuminated zones of dark–light choice nests significantly more strongly than both reverted nurses and foragers, which did not differ from each other in that respect. In all groups ants were withdrawing from the illuminated zones as a function of time. However, only nurses and foragers differed significantly from each other with respect to dynamics of dark–light choice behavior. Reverted nurses did not differ either from foragers or from nurses.

## Discussion

Our present experiment revealed that nurses of the red wood ant *Formica polyctena* avoid illuminated zones significantly more than both classes of workers that had already passed the transition from intranidal to extranidal tasks: foragers and reverted nurses. This finding implies that behavioral reversion is not accompanied by the return of illumination preferences of the reverted nurses of *F. polyctena* to the state characteristic for ordinary nurses.

Pairwise Repeated Measures ANOVA analyses discovered significant interaction “group” x “time” only in the case of the comparison of nurses with foragers. In other words, dynamics of dark–light choice differed significantly only between nurses and foragers, whereas reverted nurses did not differ with respect to that feature of their dark–light choice behavior from the remaining two groups. The dynamics of the dark–light choice behavior carried out by the reverted nurses were thus to some degree intermediate in relation to the dynamics of that behavior shown by nurses and foragers.

Differences between dark–light choice behavior of nurses and workers that had already passed the transition from intranidal to extranidal tasks confirm earlier findings obtained for *F. polyctena* and a closely akin red wood ant species *F. rufa* (Rosengren 1971, 1977; Rosengren and Sundström 1987)*.* As shown by these studies, extranidal workers avoided illuminated zones less strongly than intranidal ones during dark–light choice tests (*F. rufa*; Rosengren 1971), and older extranidal workers, the so called veterans, were more photopositive than younger extranidal workers, the so called novices (*F. polyctena;* Rosengren 1977; Rosengren and Sundström 1987). As extranidal workers of the red wood ants rely largely on visual navigation and both intensity and direction of light influence significantly their locomotory behavior (Jander 1957; Mabelis 1979; Rosengren 1971; Beugnon and Fourcassié 1988; Fourcassié and Beugnon 1988; Fourcassié 1991), decreasing avoidance of light and/or illuminated zones observed in the course of their behavioral ontogeny is not surprising. It may also be noted that honeybee foragers are also more positively phototactic than intranidal workers (Ben-Shahar et al. 2003).

Our present results can also be compared with the data obtained by Wehner et al. (1972) in his classical experiment devoted to the effect of exposure to various light conditions on behavior of intranidal and extranidal workers of the desert ant *Cataglyphis bicolor* Fabricius in the situation of choice between dark and white silhouettes. In such experimental situation intranidal workers of *C. bicolor* usually approached dark silhouettes, whereas extranidal workers preferred white ones. However, after having been exposed to light intranidal workers started rapidly to prefer white silhouettess. Foragers of *C. bicolor* exposed to darkness also reversed their preferences, but in a much slower way and even after 120 h some of them still oriented towards the white silhouettes. In our study the reverted nurses of *F. polyctena* were tested after 7 days (about 170 h) from the start of the reversion process, but, nevertheless, their illumination responses remained similar to those of foragers. However, our reverted nurses were not subjected to constant forced exposure to darkness, as in the experiment of Wehner et al. (1972), but to modifications of social context carried out to induce the reversal from extranidal tasks to intranidal brood care, and, similarly as in natural conditions, they were free to walk out of the brood chambers into the areas exposed to light. Nevertheless, reverted nurses used in our tests were taken from among individuals which stayed in artificial brood chambers covered by aluminum foil to assure darkness, whereas foragers were captured in the foraging area of their nest where they had been exposed to light. This implies that overall similarity of dark–light choice behavior of these two groups of ants was observed in spite of possible effects of short-term light adaptation, a process that had been studied in detail in *F. polyctena* (Menzel and Lange 1971; Roth and Menzel 1972; Menzel and Knaut 1973).

Behavioral differences between workers of *F. polyctena* that did/did not pass the transition from intranidal to extranidal tasks are in concordance with our present knowledge concerning age-, task- and experience-related neuroanatomical changes taking place in the visual system of ant brains (Kühn-Bühlmann and Wehner 2006; Seid et al. 2008; Stieb et al. 2010, 2012). Interestingly, Stieb et al. (2012) showed that structural changes in the synaptic organization of the calyces of the mushroom bodies accompanying the transition from intranidal to extranidal tasks in ants of are partly reversible. This result is in concordance with earlier behavioral data of Wehner et al. (1972). In the case of our present experiment the dynamics of dark–light choice behavior shown by reverted nurses also proved to be to some degree intermediate in relation to nurses and foragers. However, dark–light choice behavior of reverted nurses of *F. polyctena* remained largely similar to that of foragers, which implies that in that ant species modifications of illumination preferences accompanying the transition from intranidal to extranidal tasks are largely retained even if the individual returns back to dark intranidal environment and engages in intranidal brood care.
